# Validation of Dual Energy X-Ray Absorptiometry Measures of Abdominal Fat by Comparison with Magnetic Resonance Imaging in an Indian Population

**DOI:** 10.1371/journal.pone.0051042

**Published:** 2012-12-14

**Authors:** Amy E. Taylor, Hannah Kuper, Ravi D. Varma, Jonathan C. Wells, Jimmy D. Bell, K. V.Radhakrishna, Bharati Kulkarni, Sanjay Kinra, Nicholas J. Timpson, Shah Ebrahim, George Davey Smith, Yoav Ben-Shlomo

**Affiliations:** 1 School of Social and Community Medicine, University of Bristol, Bristol, United Kingdom; 2 MRC Centre for Causal Analyses in Translational Epidemiology, School of Social and Community Medicine, University of Bristol, Bristol, United Kingdom; 3 Department of Clinical Research, London School of Hygiene and Tropical Medicine, London, United Kingdom; 4 Krishna Institute of Medical Sciences, Hyderabad, India; 5 Childhood Nutrition Research Centre, Institute of Child Health, London, United Kingdom; 6 Metabolic and Molecular Imaging Group, MRC Clinical Sciences Centre, Imperial College, London, United Kingdom; 7 National Institute of Nutrition, Hyderabad, India; 8 Department of Non-Communicable Disease Epidemiology, London School of Hygiene and Tropical Medicine, London, United Kingdom; 9 South Asia Network for Chronic Disease, Public Health Foundation of India, New Delhi, India; University of Tor Vergata, Italy

## Abstract

**Objective:**

Abdominal adiposity is an important risk factor for diabetes and cardiovascular disease in Indians. Dual energy X-ray absorptiometry (DXA) can be used to determine abdominal fat depots, being more accessible and less costly than gold standard measures such as magnetic resonance imaging (MRI). DXA has not been fully validated for use in South Asians. Here, we determined the accuracy of DXA for measurement of abdominal fat in an Indian population by comparison with MRI.

**Design:**

146 males and females (age range 18–74, BMI range 15–46 kg/m^2^) from Hyderabad, India underwent whole body DXA scans on a Hologic Discovery A scanner, from which fat mass in two abdominal regions was calculated, from the L1 to L4 vertebrae (L1L4) and from the L2 to L4 vertebrae (L2L4). Abdominal MRI scans (axial T1-weighted spin echo images) were taken, from which adipose tissue volumes were calculated for the same regions.

**Results:**

Intra-class correlation coefficients between DXA and MRI measures of abdominal fat were high (0.98 for both regions). Although at the level of the individual, differences between DXA and MRI could be large (95% of DXA measures were between 0.8 and 1.4 times MRI measures), at the sample level, DXA only slightly overestimated MRI measures of abdominal fat mass (mean difference in L1L4 region: 2% (95% CI:0%, 5%), mean difference in L2L4 region:4% (95% CI: 1%, 7%)). There was evidence of a proportional bias in the association between DXA and MRI (correlation between difference and mean −0.3), with overestimation by DXA greater in individuals with less abdominal fat (mean bias in leaner half of sample was 6% for L1L4 (95%CI: 2, 11%) and 7% for L2L4 (95% CI:3,12%).

**Conclusions:**

DXA measures of abdominal fat are suitable for use in Indian populations and provide a good indication of abdominal adiposity at the population level.

## Introduction

Abdominal obesity is thought to be a key factor driving the current epidemic of diabetes and cardiovascular disease within India [1]. Accurate measurement of abdominal fat is important in research studies to be able to investigate its causes and consequences. Dual energy X-ray absorptiometry (DXA) is becoming a popular and widespread measure for assessment of body fat and is less costly and more accessible than the gold standard imaging methods, computed tomography (CT) and magnetic resonance imaging (MRI) [2]–[5]. Abdominal regions can be manually drawn on to whole body DXA scans, using lumbar vertebrae, the iliac crest or ribs as landmarks, to enable specific assessment of abdominal fat [6]. Although there is little DXA data available for Indian populations, there is evidence from studies in other populations that DXA abdominal regions may be more informative about metabolic risk factors (lipids, insulin, glucose) than simple anthropometry [7]–[9]. Thus, DXA abdominal measurements may prove a valuable tool for assessment of associations of adiposity with these risk factors in Indian populations.

Whilst DXA has been shown to be a robust technique for assessing of abdominal fat in Europeans [6]; [10]; [11], no direct comparisons with reference methods have been conducted in Indian populations. Some Indian studies have collected both DXA and image data, but they have not used this to validate assessment of abdominal fat. In work collecting both MRI and DXA scan data on 171 adults from North India, no DXA abdominal region of interest was selected and no direct comparison was made between DXA and MRI [12]. Abdominal regions of interest were measured by both CT and DXA in 164 participants from the Chennai Urban Rural Epidemiology Study but the two methods did not compare the same region and only correlation coefficients were used to compare the outcomes [13]. Given the differences in the type and distribution of body fat in South Asians compared to Europeans [14]; [15] and the finding that correlations between methods of body fat assessment can differ by ethnicity [4]; [16], it is important that DXA is validated against imaging methods within different populations.

Here, we tested the accuracy of DXA to estimate abdominal fat against MRI scan data in a study of 146 individuals from Hyderabad, India. We report that despite individual variations, DXA shows excellent agreement with MRI data at the population level.

## Methods

### Ethics Statement

Ethical approval was obtained from the London School of Hygiene & Tropical Medicine, the Indian Council of Medical Research, the National Institute of Nutrition and the Krishna Institute of Medical Sciences. Informed written consent was obtained from all participants.

### Study population

The study population comprised two previously studied cohorts living in the city of Hyderabad, India, and its surrounding areas. The Hyderabad arm of the Indian Migration Study (IMS) comprised rural to urban migrants and their spouses recruited from a factory in Hyderabad and their siblings who had remained in rural areas [17]. Lifelong urban factory workers and their urban siblings were also recruited. The original fieldwork for the IMS was conducted between 2005 and 2007, during which time 1995 participants were examined in Hyderabad [18]. The Hyderabad Nutrition Trial (HNT) was a study of children who were born in 29 villages on the outskirts of Hyderabad from 1987–1990. These children, their parents and siblings now form the Andhra Pradesh Children and Parents Study (APCAPS). Between 2003 and 2005, 1165 of the children (then aged 13–18) attended a research clinic [19]. From January 2009-December 2010, 2369 participants (918 IMS and 1451 HNT) attended a clinic at the National Institute of Nutrition in Hyderabad. The data presented here are cross sectional with all measures taken during the 2009–2010 clinic.

Participants were selected on the basis of predefined age, study, sex, rural/urban and BMI categories (see tables S1, S2, S3), in order to sample the entire range of BMI in the Hyderabad DXA study. The target recruitment was 160 participants with adequate scan data (100 from IMS, 60 from HNT).

### Demographic and anthropometric data

Demographic information was collected on all study participants using an interviewer administered questionnaire. Weight was measured to the nearest 0.1 kg without shoes using digital Seca scales (www.seca.com). Standing height was measured using a portable stadiometer (Leicester height measure; Chasmors Ltd, Camden, London, UK). Sitting height was measured using the same stadiometer, with the subject sitting upright on a stool of known height. Trunk length was calculated by subtracting stool height from sitting height. Waist circumference was measured twice to the nearest mm using a metallic tape measure at the narrowest point of the waist between the ribs and the iliac crest. All anthropometric measures were taken twice and the average of the two values used in the analysis. BMI was calculated as weight(kg)/height(m)^2^.

### DXA scan

Whole body DXA scans were performed on a Hologic DXA machine (Discovery A model)(www.hologic.com). During the scan, the participant was asked to lie supine on the scanning bed with their arms at their sides. The scanner was calibrated daily with a spine phantom and its performance was monitored as per quality assurance protocol.

**Figure 1:**
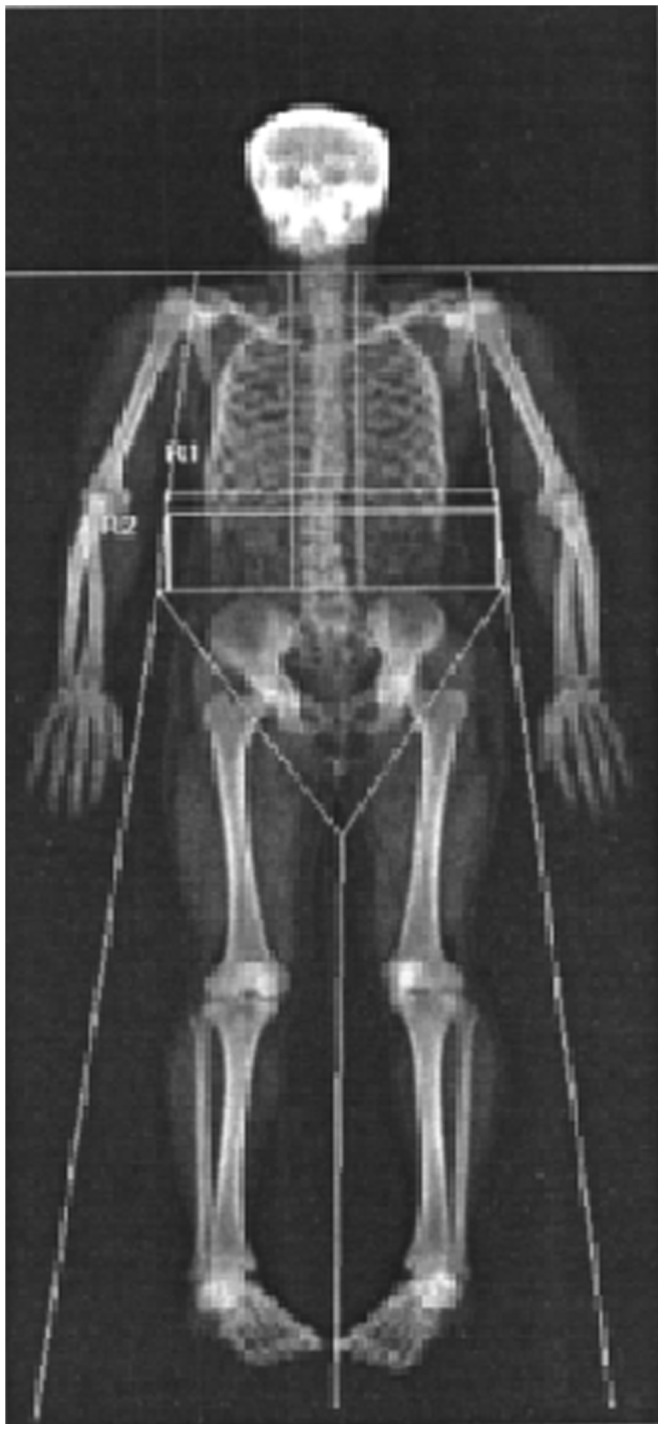
L1L4 and L2L4 regions on whole body DXA scan.

Abdominal fat measures were calculated for two regions of interest: from the midpoint of the intervertebral space between the T12 and L1 vertebrae to the midpoint of the intervertebral space between the L4 and L5 vertebrae (L1L4) and from the midpoint of the intervertebral space between the L1 and L2 vertebrae to the midpoint of the intervertebral space between the L4 and L5 vertebrae (L2L4). Both of these regions have been used in previous studies [6]; [7]; [13]; [20]. The L1L4 region has been validated against CT in a European population [6]. These regions of interest were defined by marking image areas which enclosed the abdominal regions defined by the cut points described above on to the whole body scan using the Hologic software (version 12.5) (see **Figure 1**). All analyses were performed twice for each scan by a single trained technician and the average of the repeat analyses calculated for each region. The intraclass correlation coefficients (ICCs) for repeat measures were >0.99. The outputs from the DXA scanner for the L1L4 and L2L4 regions were fat mass in grams.

### MRI scan

All abdominal MRI scans were performed within 18 days of undergoing a DXA scan (90% were performed within one week). Participants were scanned in the supine position with their arms stretched out above their heads using a GE 1.5 Tesla Signa Excite II MRI scanner. Slices of 10 mm thickness with no gaps in between were taken in the axial plane, starting at the midpoint of the L4/L5 intervertebral space, moving upwards until the T11/T12 intervertebral space. The scan parameters for the T1 weighted axial images were as follows: pulse sequence FSE Spin Echo, repetition time 640 ms, echo time 8.7 ms, band width 31.3 KHz, matrix size 256×256. Field of view (FOV) was adjusted according to the cross sectional area of the participant and ranged from 340 to 480 mm. Incomplete scans and completed scans which did not capture the entire region of interest were excluded from the analyses.

The slices corresponding to the DXA L1L4 and L2L4 regions were selected for each participant, to the nearest half slice. Scan images were analysed by Vardis (www.vardisgroup.com), using an image segmentation programme (Sliceomatic, Quebec, Canada, www.tomovision.com), which calculated volumes of subcutaneous and intra-abdominal adipose tissue in litres. The interobserver coefficients of variation for abdominal fat analysis by Vardis are: subcutaneous abdominal adipose tissue:3.6%, intra-abdominal adipose tissue:5.1%. Subcutaneous and intra –abdominal adipose tissue volumes were added together to give a total volume for each slice. Slice volumes were aggregated to give total volume of adipose tissue in the L1L4 and L2L4 regions.

### Statistical analysis

All analyses were conducted in Stata, version 11.2 (Stata corp, Texas, US). Volumes of adipose tissue from MRI were converted to mass using a conversion factor of 0.9225 kg/l, which is commonly used as the density of adipose tissue [21]–[23]. To account for the fact that adipose tissue is not wholly made up of fat but also contains water, minerals and proteins, MRI estimates were further multiplied by 0.8. This assumption of 80% fat has been used in a number of previous studies [21]; [24]; [25].

Since the study population demonstrated a wide range of adiposity, DXA and MRI fat estimates were log transformed prior to analysis so that mean differences between them could be expressed as ratios rather than absolute values. Differences between DXA and MRI (DXA minus MRI) estimates of fat mass were assessed using paired t tests. Bland Altman plots of the average of repeat measures against the difference of the two measures were constructed along with their 95% limits of agreement (mean difference ±2 standard deviations of the difference) [26]. Bias and limits of agreement were exponentiated and therefore represent ratios of DXA:MRI fat. Correlation coefficients were used to assess proportional bias (association between the average and the difference). Intra-class correlation coefficients (ICCs) were calculated from a two way analysis of variance according to the agreement definition [27]. ICCs are the ratio of the between individual variance to the total variance (between and within individuals). All analyses were conducted for the sample as a whole and additionally stratified by sex, study and amount of fat in the abdominal region (above and below the median value).

To investigate factors associated with the difference between DXA and MRI (DXA minus MRI), linear regression was performed, with the logged difference between measures as the outcome and log average fat in the region (from DXA and MRI), age, sex, study, height, trunk length, waist circumference, FOV and internal:subcutaneous fat ratio (from MRI) as potential predictors. Coefficients were expressed as ratios of geometric means and their 95% CIs.

## Results

**Figure 2:**
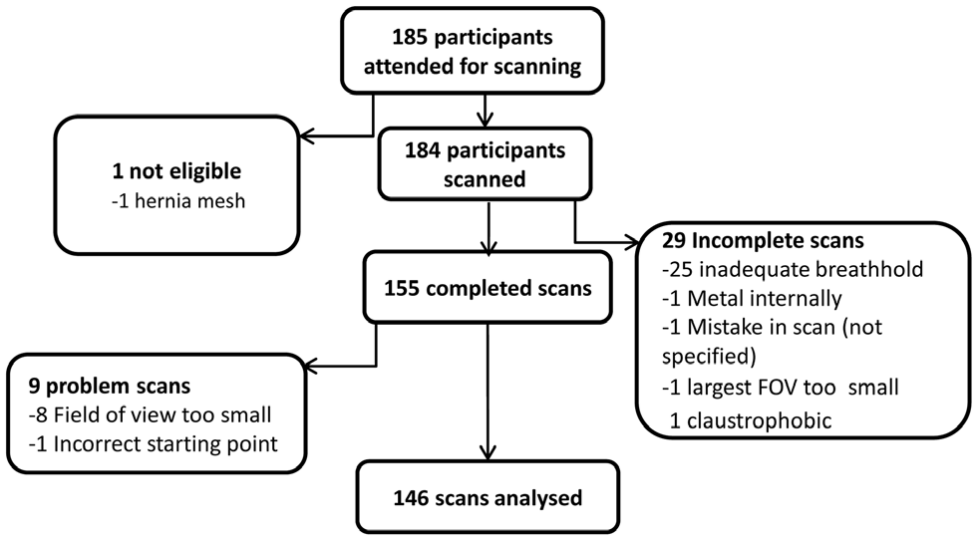
Flow chart of participants of MRI calibration study and reasons for exclusion.

Of the 185 participants who attended for MRI scanning, 146 had useable MRI scan data. Details of the reasons for exclusion are presented in **Figure 2**, but were predominantly the result of incomplete scans. A total of 59 scans were obtained from HNT participants and 87 from IMS participants (**Table 1**). Males made up 53% of the whole study sample (N = 78). The HNT participants were all aged between 19 and 23 years, with a BMI range of 14.8 to 31.0 kg/m^2^. The IMS participants were aged between 21 and 74 years with a mean age of 50 years and had a BMI range of 16.4 to 46.0 kg/m^2^. The median age of the whole study population was 42 years and the median BMI was 23.7 kg/m^2^.

**Table 1:** Characteristics of the study population.

	IMS		HNT	
	Males	Females	Males	Females
**N**	48	39	30	29
**Age (years)**	51.5 (9.2)	47.2 (6.8)	22.0 (1.1)	21.7 (1.2)
**BMI (kg/m^2^)**	25.5 (5.0)	27.3 (5.5)	21.4 (4.4)	20.1 (3.8)

IMS: Indian Migration Study, HNT: Hyderabad Nutrition Trial.

Variables presented as Mean (SD).

**Figure 3:**
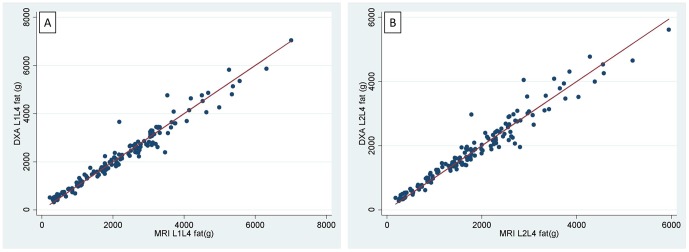
Scatter plot of DXA versus MRI fat in L1L4 and L2L4 regions. Figure 3A shows L1L4 region. Figure 3B shows L2L4 region.

In the sample as a whole, DXA estimates of L1L4 fat and L2L4 fat correlated strongly with MRI estimates (**Figure 3A and 3B**). The ICC was very high (0.98) for both regions, indicating good agreement.

**Figure 4:**
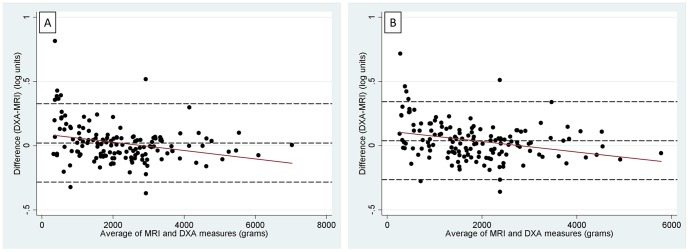
Bland Altman plots of DXA versus MRI measures of L1L4 and L2L4 fat. Figure 4A shows L1L4 region. Figure 4B shows L2L4 region. Difference between measures represented as log values. The central dashed line represents the mean difference between measures. The upper and lower dashed lines represent the 95% limits of agreement (±2SDs of the mean difference). The solid line represents the line of best fit from linear regression.

**Figure 4A** and **Figure 4B** show the Bland Altman plots of the log difference (DXA minus MRI) against the average of DXA and MRI fat mass (in grams) in the L1L4 and L2L4 regions; the corresponding statistics are shown in **Table 2**. There was evidence of a small overestimation of MRI fat mass by DXA in the sample as a whole. The ratio of DXA fat mass to MRI fat mass (mean bias) was 1.02 in the L1L4 region and 1.04 in the L2L4 region (**Table 2**). The limits of agreement showed that 95% of DXA measures of fat would be expected to be between 0.75 and 1.39 times MRI measures in the L1L4 region and between 0.77 and 1.41 times MRI measures in the L2L4 region.

**Table 2:** Bias and 95% limits of agreement for DXA measures of L1L4 and L2L4 fat compared with MRI.

L1L4	N	MRI fat (g)^1^	DXA fat (g)^1^	Bias^2^	95% CI^2^		p- value^3^	r^4^	p-value^5^	Limits of agreement^6^	
**Whole sample**	146	1737 (1536,1963)	1773(1584,1986)	1.02	(1.00,	1.05)	0.10	−0.29	<0.001	(0.75,	1.39)
**Males**	78	1773 (1479, 2126)	1761 (1490,2081)	0.99	(0.96,	1.03)	0.70	−0.28	0.01	(0.73,	1.36)
**Females**	68	1696 (1434, 2005)	1788 (1534, 2085)	1.05	(1.02,	1.09)	0.003	−0.27	0.03	(0.79,	1.41)
**IMS**	87	2571 (2327, 2841)	2524 (2282, 2792)	0.98	(0.96,	1.01)	0.14	0.06	0.60	(0.78,	1.24)
**HNT**	59	974 (808, 1174)	1054 (894,1242)	1.08	(1.03,	1.13)	0.002	−0.43	<0.001	0.75,	1.55)
**Above median** 7	73	3106 (2901,3325)	3047 (2841,3267)	0.98	(0.96,	1.01)	0.15	0.07	0.56	(0.78	1.23)
**Below median** 7	73	971 (843, 1119)	1032 (913, 1167)	1.06	(1.02,	1.11)	0.004	−0.42	<0.001	0.75,	1.51)
**L2L4**											
**Whole sample**	146	1454(1286,1643)	1510 (1350, 1690)	1.04	(1.01,	1.07)	0.003	−0.30	<0.001	(0.77,	1.41)
**Males**	78	1475 (1230,1768)	1473 (1248,1740)	1.00	(0.97,	1.03)	0.96	−0.31	0.005	(0.74,	1.35)
**Females**	68	1430 (1212, 1687)	1554 (1335,1809)	1.09	(1.05,	1.12)	<0.001	−0.27	0.03	(0.82,	1.44)
**IMS**	87	2144 (1944, 2365)	2133 (1929, 2358)	1.00	(0.97,	1.02)	0.69	0.09	0.42	(0.78,	1.28)
**HNT**	59	820 (679, 990)	908 (769, 1073)	1.11	(1.06,	1.16)	<0.001	−0.48	0.001	(0.80,	1.54)
**Above median** 7	73	2568 (2398,2750)	2579 (2409, 2761)	1.00	(0.97,	1.03)	0.77	−0.01	0.93	(0.78,	1.30)
**Below median** 7	73	823 (712, 951)	885(781, 1002)	1.07	(1.03,	1.12)	<0.001	−0.51	<0.001	(0.77,	1.50)

1 Geometric mean and 95% CI.

2 . Mean bias and 95% CI expressed as ratio of DXA:MRI values. Bias is the difference (DXA minus MRI) between log fat values from the two techniques.

3 . P value from paired t test of the difference.

4 . Correlation coefficient of the log difference between DXA and MRI against the average of DXA and MRI measures of fat mass (in grams).

5 . Significance of correlation coefficient.

6 . 95% Limits of agreement (mean difference ± 2SD) expressed as ratio of DXA:MRI values.

7 . Median average abdominal fat value (by MRI and DXA) was 2008 g for L1L4 and 1708 g for L2L4.

There was evidence of a negative correlation between the difference and the mean (r = −0.3, p<0.001 in both samples) (**Figure 4**). This negative correlation was largely driven by an overestimation of MRI fat mass by DXA at low values of abdominal fat. This is shown by the results of analyses stratified by study and by median amount of fat in the abdominal region (**Table 2**). In the L1L4 region, DXA overestimated MRI fat mass by 8% in HNT participants (95% CI: 3,13%) and by 6% (95%CI: 2, 11%) in those with below average abdominal fat values. In the IMS and the sample with below median values, DXA underestimated fat mass by 2%, but confidence intervals were consistent with no fixed mean bias in these samples. Results for the L2L4 region were similar to those for the L1L4 region. When the sample was stratified by sex, there was evidence of an overestimation of MRI fat mass by DXA in females (5% in L1L4 and 9% in L2L4) but no strong evidence for a fixed mean bias in males.

To investigate which factors were independently associated with the observed bias, multivariate linear regression was performed. The amount of fat in the abdominal region as measured by MRI was the single factor explaining most of the variance in the difference between the measures for the L1L4 region (R^2^ = 16%)(**Table 3**). A 10% increase in the amount of fat in the abdominal region was associated with a 1% decrease in the difference between measures (DXA-MRI). Associations of age and study with the difference between DXA and MRI were attenuated to the null in bivariate analysis, following adjustment for fat in the abdominal region. Multivariate analysis explained 24% of the variance in the difference between measures. The amount of fat remained negatively associated with the difference. The associations with sex and internal:subcutaneous fat ratio were attenuated and estimates were consistent with there being no associations with these variables. Each category increase in FOV was associated with a 2% increase in the difference between DXA and MRI (Coeff: 1.02, 95% CI: 1.00, 1.04). Similar results were obtained for the L2L4 region and are presented in table S4.

**Table 3:** Variables associated with the difference (DXA-MRI) between measures of abdominal fat in the L1L4 region.

	Univariate analysis				Bivariate analysis^3^				Multivariate analysis^4^			
L1L4 region	Coeff	95% CI		p	Coeff	95% CI		p	Coeff	95% CI		p
Amount of fat in L1L4 region (g)^1^	0.918	(0.889,	0.948)	<0.001					0.908	(0.876,	0.942)	<0.001
Waist circumference (cm)	0.996	(0.994,	0.998)	<0.001	1.000	(0.996,	1.003)	0.90				
Standing Height (cm)	0.998	(0.995,	1.001)	0.18	0.998	(0.996,	1.001)	0.15				
Trunk Length (cm)	0.996	(0.991,	1.001)	0.08	0.997	(0.992,	1.001)	0.22				
Sex Males	-	-		-	-	-		-	-		-	-
Females	1.062	(1.011,	1.116)	0.02	1.060	(1.014,	1.110)	0.01	1.034	(0.976,	1.094)	0.25
Age (years)	0.997	(0.996,	0.999)	0.002	1.000	(0.998,	1.002)	0.87				
Study HNT	-	-		-	-	-		-				
IMS	0.907	(0.864,	0.952)	<0.001	0.970	(0.913,	1.030)	0.31				
FOV (per category increase)^2^	1.001	(0.982,	1.020)	0.93	1.026	(1.006,	1.045)	0.009	1.021	(1.002,	1.041)	0.03
Ratio of internal: subcutaneous fat	0.907	(0.860,	0.956)	<0.001	0.932	(0.886,	0.981)	0.007	0.965	(0.904,	1.030)	0.28

Outcomes are log transformed so coefficients represent ratio of geometric means. Outcome is the difference between abdominal fat measures (DXA-MRI).

1 Average amount of fat from DXA and MRI, log transformed.

2 FOV (Field of view) categories (≤360 mm, 380 mm, 400 mm, >420 mm).

3 Adjusted for amount of fat in region.

4 Adjusted for all other variables in the model.

## Discussion

This study provides evidence that, at the population level, DXA is an appropriate measure for assessing abdominal fat in an Indian population. Our study population demonstrated a wide spectrum of adiposity, ranging from the very lean to the very obese. There was good agreement between MRI and DXA estimates of abdominal fat, with only small differences found between measures in the overall sample. However, differences between measures on the individual level are present and there was evidence of a slight proportional bias according to adiposity level, with DXA showing a tendency to overestimate MRI fat mass in the leanest individuals.

Differences at the population level between DXA and MRI measures of abdominal fat were small (<5%). The similarity of the overall mean values of abdominal fat by DXA and MRI in our study is largely consistent with results of abdominal fat MRI/CT and DXA method comparison studies conducted in European populations. Two previous studies that have compared DXA and CT measures of the L1L4 region, found that DXA estimates were in the region of 20–26% lower than CT measures [6]; [10]. These results are in concordance with those here, given that neither of these studies accounted for the composition of adipose tissue when converting between adipose tissue volume and fat mass. In work making a correction for adipose tissue composition in 148 men and women aged 70–79, it was found that DXA underestimated CT assessed adiposity by about 10% [25]. However, they only performed a single slice CT and multiplied this up to equate to the same volume as the DXA region, making the assumption that adipose tissue volume was constant across the region. It has been shown that single slice imaging methods are unlikely to accurately quantify fat in the entire abdominal region [28].

Jensen et al compared DXA measures of the same abdominal regions against CT in 21 individuals and found little evidence of differences between measures [11]. Their region of interest differed from ours, as it was measured from the dome of the diaphragm to the top of the femur. However, given that they only corrected for the density of triglycerides (0.9 kg/L) when converting DXA fat mass in to CT volume and not for the fat content of adipose tissue, an underestimation of CT by DXA would be expected. The authors point to exclusion of adipose tissue in the CT analysis and problems with the assumption of triglyceride density as possible reasons for the lack of difference [11]. In addition, whilst most fat in the body is located in adipose tissue, there is fat present in other tissues, such as muscle and the liver, which is captured by DXA but not by imaging methods [29].

Whilst there was little overall fixed bias between DXA and MRI estimates of abdominal fat, we found that DXA tended to overestimate abdominal fat mass in leaner individuals. Bias by subject size is a common feature of method comparison studies of DXA measures of body fat [25]; [30]–[33] and has been shown to act in the opposite direction (DXA underestimating fat in leaner individuals and overestimating in fatter individuals) in several studies [30]–[32]. Comparison of our results with these studies is difficult due to the use of different DXA machines, different reference methods, different age groups and different scan regions (abdominal vs whole body). However, it is possible that body composition differences in Indians compared to Europeans, e.g. relative differences in fat and lean mass [34], may affect the direction of these biases.

There was a mean overestimation of 6% of MRI by DXA for L1L4 fat in the leaner half of the sample and wide limits of agreement (0.75 to 1.51 times MRI values).One possible explanation is the partial volume phenomenon; since individual MRI pixels can only be classified as either fat or lean, misclassification of fat tissue may be greater in leaner individuals, who have more pixels which contain both fat and lean tissue [25]. Some support for this was shown in linear regression analysis; after adjustment for the amount of fat in the abdominal region, there was some indication that size of field of view (FOV) was associated with the difference between DXA and MRI. As FOV increases, pixel size increases, so large pixel sizes in lean individuals could lead to greater misclassification of fat tissue. Overestimation of abdominal fat in the leanest individuals would serve to reduce the variance of the true abdominal fat values in the population when measured by DXA. This could result in differences in the magnitude of observed associations of DXA measures of abdominal fat with outcomes or exposures in epidemiological studies compared to MRI measures.

There are several limitations to this study. The target recruitment for the MRI study (N = 160) was not achieved. However, participants represented the range of BMIs in the study as a whole and the final sample size (N = 146) was comparable to or larger than most previous studies comparing MRI/CT and DXA [6]; [10]. We were unable to perform repeat DXA or MRI scans, so were unable to account for variation within each method. Poor within method repeatability is likely to result in poor agreement between methods [35]. Both DXA and MRI have been shown to be reasonably precise measures of body fat [36]; [37], although estimation of fat in the trunk region (which includes the abdomen) by DXA has shown greater variability than in other regions [38]. Estimates of body composition have been shown to differ by DXA scanner manufacturer, model and even software version [39]–[41], so these results cannot be generalised to all DXA scanners. DXA does expose participants to a low dose of radiation and whilst it is regarded as safe for most individuals, it is not recommended for use in pregnant women [42].

Comparisons between methods which measure different components of body composition (chemical fat vs adipose tissue) are complex and there will always be some error introduced in converting between them. We used a commonly reported estimate of the fat content of adipose tissue (80%) for our comparison [21]; [24]; [25] but there is a wider range reported in the literature (50–90%) [43]. It has also been reported that the estimated fraction of fat in human adipose tissue is positively associated with overall body fatness [44]; [45], but we felt that adjustment for this in our study was inappropriate given that these findings are based on small studies in individuals who were not of South Asian origin.

Bearing in mind the caveat that DXA estimates of MRI abdominal fat can be subject to large differences at the individual level, the results of this study provide good evidence that DXA scans are suitable for measuring abdominal fat in Indians at the population level. DXA measures are likely to provide good proxies for MRI measured adipose tissue in epidemiological studies. Further validation studies in South Asian populations would be useful to investigate whether DXA consistently overestimates abdominal fat mass in lean individuals.

## Supporting Information

Table S1 **Total numbers of analysed scans from the Nutrition Trial by original recruitment criteria (N = 59)**.
(DOCX)

Table S2 **Total numbers of analysed scans from IMS males by original recruitment criteria (N = 48)**.
(DOCX)

Table S3 **Total numbers of analysed scans from IMS females by original recruitment criteria (N = 39)**.
(DOCX)

Table S4 **Variables associated with the difference between DXA and MRI measures of abdominal fat in the L2L4 region**.
(DOCX)
