# Supplementary material for: Validation of Dual Energy X-Ray Absorptiometry Measures of Abdominal Fat by Comparison with Magnetic Resonance Imaging in an Indian Population
Source: PLoS One. 2012 Dec 14;7(12):e51042. doi: 10.1371/journal.pone.0051042 (PMC3522679; doi:10.1371/journal.pone.0051042)
Supplement: Table S1 — Total numbers of analysed scans from the Nutrition Trial by original recruitment criteria (N = 59). (DOCX) [file pone.0051042.s001.docx]

*Table S1. Total numbers of analysed scans from the Nutrition Trial by original recruitment criteria (N=59)*

|  | Intervention arm | | | | | | | | Control arm | | | | | | | |
| --- | --- | --- | --- | --- | --- | --- | --- | --- | --- | --- | --- | --- | --- | --- | --- | --- |
|  | Males | | | | Females | | | | Males | | | | Females | | | |
| BMI category | <17 | 17-20.99 | 21-24.99 | ≥25 | <16.5 | 16.5-19.49 | 19.5-21.99 | ≥22.0 | <17 | 17-20.99 | 21-24.99 | ≥25 | <16.5 | 16.5-19.49 | 19.5-21.99 | ≥22.0 |
| No. planned:  No. completed: | 4  **4** | 4  **4** | 4  **4** | 3  **3** | 4  **3** | 4  **4** | 4  **4** | 3  **3** | 3  **3** | 4  **4** | 4  **4** | 4  **4** | 3  **3** | 4  **4** | 4  **4** | 4  **4** |
