# Supplementary material for: Validation of Dual Energy X-Ray Absorptiometry Measures of Abdominal Fat by Comparison with Magnetic Resonance Imaging in an Indian Population
Source: PLoS One. 2012 Dec 14;7(12):e51042. doi: 10.1371/journal.pone.0051042 (PMC3522679; doi:10.1371/journal.pone.0051042)
Supplement: Table S2 — Total numbers of analysed scans from IMS males by original recruitment criteria (N = 48). (DOCX) [file pone.0051042.s002.docx]

*Table S2. Total numbers of analysed scans from IMS males by original recruitment criteria (N=48)*

| Males | | | | | | | |
| --- | --- | --- | --- | --- | --- | --- | --- |
| Rural non migrants | | | | Urban (migrants and non migrants) | | | |
|  |  | Age<50 | Age 50+ |  | | Age<50 | Age 50+ |
| BMI |  |  |  | BMI |  |  |  |
| <20 | No. Planned  No. completed | 2  **1** | 3  **1** | <23 | No. Planned  No. completed | 4  **7** | 3  **6** |
| 20-23.9 | No. Planned  No. completed | 3  **0** | 2  **0** | 23-25.9 | No. Planned  No. completed | 4  **7** | 4  **6** |
| 24-27.9 | No. Planned  No. completed | 2  **1** | 3  **1** | 26-29.9 | No. Planned  No. completed | 4  **1** | 4  **7** |
| ≥28 | No. Planned  No. completed | 3  **0** | 2  **0** | ≥30 | No. Planned  No. completed | 3  **1** | 4  **9** |
