# Supplementary material for: Validation of Dual Energy X-Ray Absorptiometry Measures of Abdominal Fat by Comparison with Magnetic Resonance Imaging in an Indian Population
Source: PLoS One. 2012 Dec 14;7(12):e51042. doi: 10.1371/journal.pone.0051042 (PMC3522679; doi:10.1371/journal.pone.0051042)
Supplement: Table S3 — Total numbers of analysed scans from IMS females by original recruitment criteria (N = 39). (DOCX) [file pone.0051042.s003.docx]

*Table S3. Total numbers of analysed scans from IMS females by original recruitment criteria (N=39)*

| Females | | | | | | | |
| --- | --- | --- | --- | --- | --- | --- | --- |
| Rural non migrants | | | | Urban (migrants and non migrants) | | | |
|  |  | Age<50 | Age 50+ |  | | Age<50 | Age 50+ |
| BMI |  |  |  | BMI |  |  |  |
| <20 | No. Planned:  No. Completed: | 2  **0** | 3  **0** | <24 | No. Planned:  No. Completed: | 4  **4** | 3  **2** |
| 20-23.9 | No. Planned:  No. Completed: | 3  **4** | 2  **2** | 24-27.9 | No. Planned:  No. Completed: | 4  **5** | 4  **2** |
| 24-27.9 | No. Planned:  No. Completed: | 2  **2** | 3  **1** | 28-32.9 | No. Planned:  No. Completed: | 4  **7** | 4  **4** |
| ≥28 | No. Planned:  No. Completed: | 3  **1** | 2  **0** | ≥33 | No. Planned:  No. Completed: | 3  **3** | 4  **2** |
