# Supplementary material for: Validation of Dual Energy X-Ray Absorptiometry Measures of Abdominal Fat by Comparison with Magnetic Resonance Imaging in an Indian Population
Source: PLoS One. 2012 Dec 14;7(12):e51042. doi: 10.1371/journal.pone.0051042 (PMC3522679; doi:10.1371/journal.pone.0051042)
Supplement: Table S4 — Variables associated with the difference between DXA and MRI measures of abdominal fat in the L2L4 region. (DOCX) [file pone.0051042.s004.docx]

*Table S4. Variables associated with the difference between DXA and MRI measures of abdominal fat in the L2L4 region*

|  | **Univariate analysis** | | | | **Bivariate analysis^3^** | | | | **Multivariate analysis^4^** | | | |
| --- | --- | --- | --- | --- | --- | --- | --- | --- | --- | --- | --- | --- |
| **L2L4 region** | **Coeff** | **95% CI** |  | **p** | **Coeff** | **95% CI** |  | **p** | **Coeff** | **95% CI** |  | **p** |
| Amount of fat in L2L4 region (g)^1^ | 0.917 | (0.888, | 0.947) | <0.001 |  |  |  |  | 0.907 | (0.876, | 0.939) | <0.001 |
| Waist circumference (cm) | 0.996 | (0.994, | 0.997) | <0.001 | 0.998 | (0.995, | 1.001) | 0.30 |  |  |  |  |
| Standing Height (cm) | 0.998 | (0.995, | 1.001) | 0.16 | 0.998 | (0.996, | 1.001) | 0.12 |  |  |  |  |
| Trunk Height (cm) | 0.995 | (0.990, | 1.000) | 0.06 | 0.996 | (0.992, | 1.001) | 0.11 |  |  |  |  |
| Sex Males |  |  |  | - | - | - |  | - | - |  | - | - |
| Females | 1.088 | (1.037, | 1.141) | 0.001 | 1.089 | (1.042, | 1.137) | <0.001 | 1.051 | (0.998, | 1.107) | 0.06 |
| Age (years) | 0.997 | (0.995, | 0.999) | <0.001 | 0.999 | (0.997, | 1.001) | 0.32 |  |  |  |  |
| Study HNT |  |  |  |  |  |  |  |  |  |  |  |  |
| IMS | 0.898 | (0.856, | 0.941) | <0.001 | 0.953 | (0.898, | 1.011) | 0.106 |  |  |  |  |
| FOV (per category increase)^2^ | 1.005 | (0.986, | 1.024) | 0.62 | 1.031 | (1.012, | 1.050) | <0.001 | 1.024 | (1.006, | 1.043) | 0.009 |
| Ratio of internal: subcutaneous fat | 0.879 | (0.834, | 0.926) | <0.001 | 0.902 | (0.858, | 0.949) | <0.001 | 0.945 | (0.889, | 1.005) | 0.07 |

Outcomes are log transformed so coefficients represent ratio of geometric means. Outcome is the difference between abdominal fat measures (DXA-MRI).

1 Average amount of fat from DXA and MRI, log transformed

2 FOV (Field of view) categories (≤360mm, 380mm, 400mm, >420mm)

3 Adjusted for amount of fat in region

4 Adjusted for all other variables in the model
